# Nrf2/Keap1/ARE Signaling Mediated an Antioxidative Protection of Human Placental Mesenchymal Stem Cells of Fetal Origin in Alveolar Epithelial Cells

**DOI:** 10.1155/2019/2654910

**Published:** 2019-05-14

**Authors:** Xiurui Yan, Xue Fu, Yuanyuan Jia, Xiaona Ma, Jin Tao, Tingting Yang, Haibin Ma, Xueyun Liang, Xiaoming Liu, Jiali Yang, Jun Wei

**Affiliations:** ^1^College of Clinical Medicine, Ningxia Medical University, Yinchuan, Ningxia 750004, China; ^2^Institute of Human Stem Cell Research, General Hospital of Ningxia Medical University, Yinchuan, Ningxia 750004, China; ^3^Clinical Laboratory Center, Institute of Hematology and Blood Disease Hospital, Chinese Academy of Medical Sciences and Peking Union Medical College, Tianjin 300020, China; ^4^College of Life Science, Ningxia University, Yinchuan, Ningxia 750021, China; ^5^Ningxia Key Laboratory of Clinical and Pathological Microbiology, General Hospital of Ningxia Medical University, Yinchuan, Ningxia 750004, China

## Abstract

The oxidative stresses are a major insult in pulmonary injury such as acute lung injury (ALI) and acute respiratory distress syndrome (ARDS), two clinical manifestations of acute respiratory failure with substantially high morbidity and mortality. Mesenchymal stem cells (MSCs) hold a promise in treatments of many human diseases, mainly owing to their capacities of immunoregulation and antioxidative activity. The strong immunoregulatory role of human placental MSCs of fetal origin (hfPMSCs) has been previously demonstrated; their antioxidant activity, however, has yet been interrogated. In this report, we examined the antioxidative activity of hfPMSCs by accessing the ability to scavenge oxidants and radicals and to protect alveolar epithelial cells from antioxidative injury using both a cell coculture model and a conditioned culture medium (CM) of hfPMSCs. Results showed a comparable antioxidative capacity of the CM with 100 *μ*M of vitamin C (VC) in terms of the total antioxidant capacity (T-AOC), scavenging abilities of free radicals DPPH, hydroxyl radical (·OH), and superoxide anion radical (O_2_^−^), as well as activities of antioxidant enzymes of SOD and GSH-PX. Importantly, both of the CM alone and cocultures of hfPMSCs displayed a protection of A549 alveolar epithelial cells from oxidative injury of 600 *μ*M hydrogen peroxide (H_2_O_2_) exposure, as determined in monolayer and transwell coculture models, respectively. Mechanistically, hfPMSCs and their CM could significantly reduce the apoptotic cell fraction of alveolar epithelial A549 cells exposed to H_2_O_2_, accompanied with an increased expression of antiapoptotic proteins Bcl-2, Mcl-1, Nrf-2, and HO-1 and decreased proapoptotic proteins Bax, caspase 3, and Keap1, in comparison with naïve controls. Furthermore, hfPMSCs-CM (passage 3) collected from cultures exposed an inhibition of the Nrf2/Keap1/ARE signaling pathway which led to a significant reduction in caspase 3 expression in A549 cells, although the addition of Nrf2 inhibitor ML385 had no effect on the antioxidative activity of hfPMSCs-CM. These data clearly suggested that hfPMSCs protected the H_2_O_2_-induced cell oxidative injury at least in part by regulating the Nrf2-Keap1-ARE signaling-mediated cell apoptosis. Our study thus provided a new insight into the antioxidative mechanism and novel functions of hfPMSCs as antioxidants in disease treatments, which is warranted for further investigations.

## 1. Introduction

The imbalance between oxidant and antioxidant is mainly caused by the increased production of reactive oxygen species (ROS), which may result in oxidative stress in the body. As a consequence, the oxidative stress usually leads to cell necrosis and apoptosis and ultimately causes systemic inflammatory responses and diseases including chronic heart failure [1], Alzheimer's disease [2], atherosclerosis, diabetes [3], systemic lupus erythematosus (SLE), and cancer [4]. In this regard, an oxidative injury has been recognized as a major inducer of pulmonary injury, which is associated with the development and progression of acute lung injury (ALI) and acute respiratory distress syndrome (ARDS). Despite that huge progresses have been made in the treatments of these diseases in the past few decades, the mortality rate of ALI/ARDS remains approximately 40% [5]. Therefore, new effective therapeutic agents and/or strategies are urgently required for ALI/ARDS treatments.

Mesenchymal stem cells (MSCs) have potencies of proliferation, differentiation, self-renewal, and a capacity of differentiation into multiple cell types/lineages such as adipocytes, osteoblasts, chondrocytes, and neurocytes *in vivo* and *in vitro* [6]. In general, MSCs can be isolated from various tissues, such as bone marrow (BM), adipose tissue, and placenta [7]. In this regard, fetal placental mesenchymal stem cells (fPMSCs) have been shown higher characteristics of proliferation, stemness, differentiation, and immunomodulation than other MSCs isolated from adult tissues or organs [8, 9]. Functionally, MSCs can exert their functions by secreting secretomes, which include chemokines, cytokines, growth factors, and extracellular vesicles (EVs). To date, MSCs as well as the MSC secretome derived from distinct origins of tissues have been tested and/or applied in treatments of many diseases in clinical trials, mainly owing to their immunoregulatory roles [10–13]. Previous studies on ARDS have shown that MSCs have antioxidative stress properties [14]. For example, Shalaby and colleagues found that MSCs could alleviate lung injury and increase the activity of antioxidant enzymes in serum of rat ALI caused by *Escherichia coli* suspension [14]. Similarly, an *in vitro* study by Park and coworkers also revealed that a conditioned medium (CM) derived from fPMSCs could effectively reduce the expression of muscle atrophy-related proteins in myocytes, inhibit the production of ROS, and increase the expression of antioxidant enzymes. Mechanistically, recently studies have demonstrated that the nuclear factor erythroid-derived 2-like 2- (Nrf2-) Kelch-like ECH-associated protein 1- (keap1-) antioxidant response element (ARE) signaling pathway is one of the most important cellular defense mechanisms against oxidative stress [15, 16]. In this respect, MSCs modified with heme oxygenase-1 (HO-1) could enhance paracrine production of hepatocyte growth factor (HGF), interleukin- (IL-) 10, and the activity of Nrf2 to attenuate lipopolysaccharide- (LPS-) induced oxidative damage in pulmonary microvascular endothelial cells (PVECs) [16]. In addition, the marrow mesenchymal stem cell- (BMSC-) mediated alleviation of bleomycin-induced pulmonary fibrosis was found through a mechanism by activating the HO-1 expression and the Nrf2 pathway [15]. However, the underlying mechanism by which the secretome of hfPMSC attenuated the degree of ALI has not been fully understood.

We have recently shown that the hfPMSC showed a significant function in promoting angiogenesis *in vitro* and increasing an immunosuppressive function *in vivo* by expressing express HGF and CD200 [17]. Interestingly, fPMSC (from passage 3 to passage 8) during long-term culture under serum-free conditions represents the detection of genetic and/or epigenetic alterations [18]. In view of aforementioned studies, together with our previous findings in the immunoregulatory roles of human placental mesenchymal stem cells of fetal origin (hfPMSCs) [17–19], we hypothesize that both of the hfPMSCs and their derived conditioned medium (CM) may have antioxidative potencies and are able to protect lung epithelial cell injury from oxidative stresses.

## 2. Materials and Methods

### 2.1. Ethics Statement

The study and protocol were approved by the ethics committee for conduction of human research at General Hospital of Ningxia Medical University (NXMU-2016-063). All healthy mothers gave written informed consent for the collection and use of placentas. Human full-term placentas were obtained from women undergoing natural delivery or caesarean section in the General Hospital of Ningxia Medical University, Yinchuan, China.

### 2.2. Isolation and Culture of hfPMSCs *In Vitro* Using a Serum-Free Medium

hfPMSCs from nine human full-term placental tissues were tested in this study. The isolation of fPMSCs was carried out and described in our previous studies [17–19]. The hfPMSCs were cultured in a serum-free medium composed of MesenCult®-XF Basal Medium containing MesenCult®-XF Supplement (STEMCELL Technologies Inc., Grenoble, France), supplemented with 50 *μ*g/mL of gentamicin (Invitrogen, Carlsbad, CA, USA). The cells were maintained in the culture medium at 37°C in a humidified environment with 5% CO_2_. For cell expansion culture, cells were dissociated and passaged using the MesenCult-ACF Dissociation Kit (STEMCELL Technologies Inc., Grenoble, France) when cells reached a confluence of about 90%. The hfPMSCs (from passages 2 to 6) were seeded at 1 × 10^6^ cells/100 mm dish with 10 mL medium and cultured for 48 hours. The culture media were then collected and clarified by centrifugation at 2000×g for 10 minutes at 4°C. Then, the hfPMSC-conditioned medium (hfPMSC-CM) was frozen at -80°C till used.

### 2.3. Oxidative Injury of A549 Alveolar Epithelial Cells

The cell viability was detected with TransDetect Cell Counting Kit-8 (CCK-8) (Transgen Biotech, Beijing, China). A549 cells were seeded into 96-well plates at a density of 5 × 10^3^ per well in a 100 *μ*L medium and cultured for 12 hours, prior to being treated with different concentrations of H_2_O_2_ (200, 400, 500, 600, and 800 *μ*mol/L) for an additional 6, 12, and 24 hours, respectively. Then, 10 *μ*L (40 g/L) of CCK-8 reagent was added to each well and incubated at 37°C for another 2 hours. The cell viability was then determined by the readout of a microplate spectrophotometer at 450 nm (Bio-Rad Laboratories Inc., Hercules, CA, USA). The mean of five wells was collected in one experiment for each point. The optimal condition (H_2_O_2_ concentration and stimulating duration) for oxidative injury was determined when 50% of cell survival was reached.

### 2.4. Flow Cytometry Assay for Cell Apoptosis

A549 cells were seeded into 6-cell culture plates (3 × 10^6^/well) for 12 h prior to being treated with 600 *μ*mol/L H_2_O_2_ for 24 h for induction of oxidative injury. The culture medium was replaced with 100 *μ*mol/L of Vitamin C (VC) or hfPMSCs-CM, and the cells were incubated for an additional 12 h before they were used for analysis. For flow cytometry assay (FACS), the cells were detached and labeled using an Annexin V-FITC/propidium iodide (PI) apoptosis detection kit per the manufacturer's instruction (BD Biosciences, San Jose, CA, USA). Briefly, cells (1 × 10^5^) in each group were incubated in a 100 *μ*L binding buffer with 5 *μ*L Annexin V-FITC and 5 *μ*L PI for 15 minutes at room temperature (RT). After incubation, 1.5 mL binding buffer was added in each tube and the cells were resuspended in PBS. Apoptotic and necrotic cells were quantified using a flow cytometer (BD FACSCalibur, San Jose, CA, USA) and analyzed in the CellQuest software. At least 10,000 cells were analyzed for each sample. Cells negative for Annexin V and PI were considered viable; cells stained with Annexin V^+^/PI^−^ were indicative of early apoptosis, whereas Annexin V^+^/PI^+^ cells were considered as late apoptosis and necrotic cell populations.

### 2.5. Hoechst 33258 Staining Assay

The cell apoptosis also was evaluated by Hoechst 33258 staining. The A549 cells (1.5 × 10^5^/well) were seeded into 6-well plates and treated with or without H_2_O_2_ (600 *μ*mol/L) for 24 hours. Then, cells were fixed and permeabilized with 0.1% formaldehyde and 0.2% Triton X-100 at RT for 10 min, respectively. After rinsing three times with 1× PBS for 5 min each, the cell nuclei were stained with Hoechst 33258 solution (5 mg/L) for 5 min at RT. The images were acquired under a fluorescence microscope (OLYMPUS, Tokyo, Japan). Cells were scored apoptotic if the nuclei presented chromatin condensation, marginalization, or nuclear beading.

### 2.6. Coculture of hfPMSCs and A549 Cells

A noncontact coculture of hfPMSCs and A549 alveolar epithelial cells was established using 6-well Millicell inserts (0.4 mm pore, polycarbonate, Millipore, Billerica, MA, USA). For injury repair (group 1), A549 cells (3.0 × 10^6^/well) were seeded in the lower chamber of a 6-well plate and cultured for 12 h prior to being treated with 600 *μ*mol/L H_2_O_2_ for 24 h. The injured cells were refreshed with 100 *μ*mol/L VC or coculture with hfPMSCs (3.0 × 10^6^/well) that were precultured on the apical membrane of Millicell inserts, and the cells were cocultured for an additional 12 h before they were used for analysis. For protection from oxidative injury (group 2), A549 cells (3.0 × 10^6^/well) were seeded in the lower chamber of a 6-well plate and cultured for 12 h prior and added with 100 *μ*mol/L VC or coculture with hfPMSCs (3.0 × 10^6^/well) for 24 h. Then, the medium of the bottom chamber was replaced with a regular culture medium containing 600 *μ*mol/L H_2_O_2_ and cultured for an additional 24 h.

### 2.7. Western Blot Analysis

Western blotting assay was utilized to analyze the expressions of proteins related to apoptosis and Nrf2-Keap1-ARE signaling in A549 cells *in vitro*. Total protein was extracted from cells using a cell lysis buffer (Biyuntian Biotech, Beijing, China). Cells were rinsed with pre-cold PBS at 4°C, with a RIPA cell lysis buffer (50 mM Tris-HCl, pH 7.5, 5 mM EDTA, 150 mM NaCl, and 0.5% NP-40) then added, and kept on ice for 30 min. Then, the cell lysate was collected by centrifugation at 12,000×g for 15 min at 4°C. The resultant supernatants were collected as whole cell extracts. The concentration of protein was detected using BCA Protein Assay Kit (BOSTER, Wuhan, China). The crude cell extract (50 *μ*g) was resolved by a 12% sodium dodecyl sulfate- (SDS-) polyacrylamide gel (SDS-PAGE) and then transferred onto a polyvinylidene fluoride (PVDF) membrane (Millipore, Billerica, MA, USA). The transferred membranes were blocked in 5% nonfat milk for 1 hour at RT and probed with rabbit anti-B cell leukemia-2(Bcl2), caspase 3, BCL2-associated X (Bax), nuclear factor (erythroid-derived 2)-like 2 (Nrf2), Keap1, heme oxygenase-1 (HO-1), beta-actin antibody (Proteintech China, Wuhan, China), and rabbit anti-Mcl-1 (Boster, Wuhan, China) at 4°C overnight. The membranes were washed with TBST three times and subsequently incubated with horseradish peroxidase-conjugated goat anti-rabbit secondary antibodies (Thermo Fisher, Waltham, MA, USA) for 2 hr at RT. The abundance of proteins of interest was visualized using the enhanced chemiluminescence (ECL) reagent (Advansta, Menlo Park, CA, United States). The relative abundance of protein was semiquantified by optical densitometry using ImageJ Software version 1.46 (https://rsb.info.nih.gov/ij/). The relative ratio of the net intensity of each sample normalized by the *β*-tubulin internal control was calculated as densitometric arbitrary units (A.U.), which was served as an index of relative expression of the protein of interest.

### 2.8. Additional Reagents and Materials

The Total Antioxidative Capacity (T-AOC) Detection Kit, Hydroxyl Free Radical (·OH) Detection Kit, Superoxide Anion Free Radical (O) Detection Kit, Superoxide Dismutase (SOD) Detection Kit, and Glutathione Peroxidase (GSH-PX) Detection Kit were purchased from Nanjing Jiancheng Bioengineering Institute (Nanjing, Jiangsu, China). The DPPH Kit for free radical scavenging ability was purchased from Sigma-Aldrich (St. Louis, MO, USA). All these kits were used to detect the antioxidative capacity of hfPMSC-CMs from different passages.

### 2.9. Statistical Analysis

The data were presented as means ± SD (standard deviation) of at least three independent experiments for each condition. Statistical analysis was performed using SPSS 17.0 analysis software (SPSS Inc., Chicago, IL, USA) and/or PRISM 5 (GraphPad software, La Jolla, CA, USA). Statistical evaluation of the data was performed by a *t*-test for comparison of differences between two groups. Significant differences were assigned to *p* values < 0.05 and < 0.01 and denoted by ∗ and ∗∗, respectively.

## 3. Results

### 3.1. Antioxidative Capacity of the hfPMSC-Conditioned Medium (CM)

**Figure 1:**
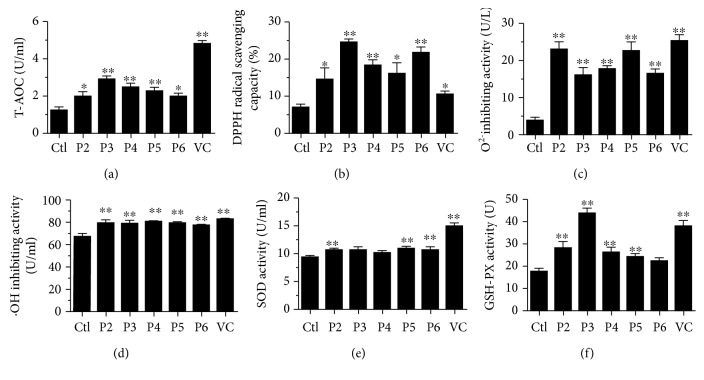
Antioxidant capacity of culture supernatants from different passages of MSCs. Alterations of T-AOC (a), DPPH radical scavenging capacity (b), O_2_ inhibiting activity (c), •OH inhibiting activity (d), SOD activity (e), and GSH-PX activity (f) in different hfPMSC-conditioned media vs. control medium. Data represented the mean ± SD from three independent triplicated experiments (*N* = 9, *t*-test). ∗ and ∗∗ represent *p* < 0.05 and *p* < 0.01, respectively.

In order to assess the antioxidative capacity of the hfPMSC-conditioned medium (hfPMSCs-CM) from passage 2 (P2) to P6, the total antioxidant capacity (T-AOC), free radical scavenging ability of DPPH, the inhibitory ability to hydroxyl radical (·OH), superoxide anion radical (O_2_^−^), and antioxidant enzyme activity (superoxide dismutase and glutathione peroxidase) were evaluated. In addition, vitamin C (VC) (100 *μ*mol/L) (see Supplementary Figure 1A), a natural antioxidant able to regulate the balance between cell proliferation and apoptosis [20], was employed as a positive control of antioxidant in this study. As shown in Figure 1(a), the T-AOC was significantly higher in hfPMSC-CM from P2 to P6 than a naïve control medium was. Of note, the highest T-AOC of hfPMSC-CM was found in P3 cells. The T-AOC of CM from P2 to P6 was comparable with 40-60 mol/L VC (*p* < 0.05) (see Supplementary Figure 1B). This result implied that hfPMSCs-CM, especially in the CM from P3 cells, had a comparable antioxidant activity with 100 *μ*mol/L of VC (Figure 1).

To further explore the antioxidative capacity of hfPMSC-CM, the capacity of CM to scavenge several oxidant radicals and activity of antioxidant enzymes was also examined. Results of radical scavenging assay showed that the free radical DPPH was significantly scavenged by hfPMSC-CM of P3-P6 cells than the control group was (Figure 1(b)). The superoxide anion radical (O_2_^−^) and hydroxyl radical (·OH) were also significantly inhibited by hfPMSC-CM, as compared to the naïve fresh control medium (*p* < 0.01) (Figures 1(c) and 1(d)). The assay of superoxide dismutase (SOD) activity also revealed a higher SOD activity in hfPMSC-CM from P2, P5, and P6 cells over the control (*p* < 0.01) (Figure 1(e)). Similarly, a higher glutathione peroxidase (GSH-PX) activity was also detected in CM from P2, P3, P4, and P5 cell cultures compared to the control medium (*p* < 0.01) (Figure 1(f)). These results suggested that hfPMSC-CM had an antioxidative capacity and scavenging ability of free radicals.

### 3.2. H_2_O_2_ Causes Oxidative Injury and Induces Apoptosis of A549 Cells *In Vitro*

**Figure 2:**
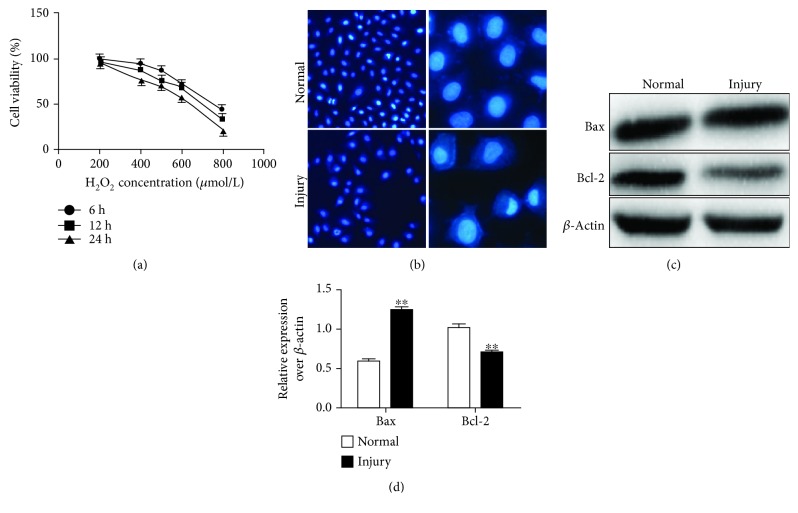
Effects on H_2_O_2_-induced oxidative injury in A549 cells *in vitro*. (a) The survival curve of A549 cells exposed to different concentrations of H_2_O_2_. (b) Hoechst 33258 staining for the nucleus of A549 cells revealed that A549 cells in the injury group had different degrees of apoptosis. (c) Immunoblotting assay confirmed the apoptosis relative protein expression of Bax and Bcl2. D. Semiquantitative analysis of the fold changes of the expression of proteins in (c) accessed by a densitometric assay. Data represented the mean ± SD from three independent triplicated experiments (*N* = 9, *t*-test). Compared with the untreated group, ∗∗ represents *p* < 0.01.

Since the adenocarcinoma cell line A549 cells were found to maintain decent properties of alveolar epithelial cells to differentiate from alveolar epithelial cells by expressing AECII markers in an air-liquid interface (ALI) state, which are able to express AECII markers and partially mimic the property of alveolar epithelia *in vitro*, the A549 cells were used with alveolar epithelial cells in the study [21]. In order to uncover the cell-protective capacity of hfPMSC-CM from oxidative injury, A549 alveolar epithelial cells were injured with different concentrations of H_2_O_2_. The results showed an exposure of 600 *μ*mol/L of H_2_O_2_ for 24 hours; the survival rate of A549 cells was reduced to 56.41 ± 3.31% (Figure 2(a)). Therefore, this treatment was employed as the oxidative injury for A549 in this study. Next, we sought to detect the influence of 600 *μ*mol/L H_2_O_2_ in cell apoptosis. Indeed, the result of Hoechst 33258 staining showed that the nucleus of A549 cells in the injury group had various degrees of cell apoptosis (condensed, compact morphology and granular nuclear fluorescence), while cells in the untreated group exhibited normal nuclear morphology (Figure 2(b)). Immunoblotting assay further demonstrated that proapoptotic protein Bax was more abundant, while the expression of antiapoptotic gene Bcl2 was significantly decreased in cells of the injury group compared to the control group (Figures 2(c) and 2(d)) (*p* < 0.01).

### 3.3. hfPMSC-CM Protected an H_2_O_2_-Induced Cell Oxidative Injury by Inhibiting Cell Apoptosis

**Figure 3:**
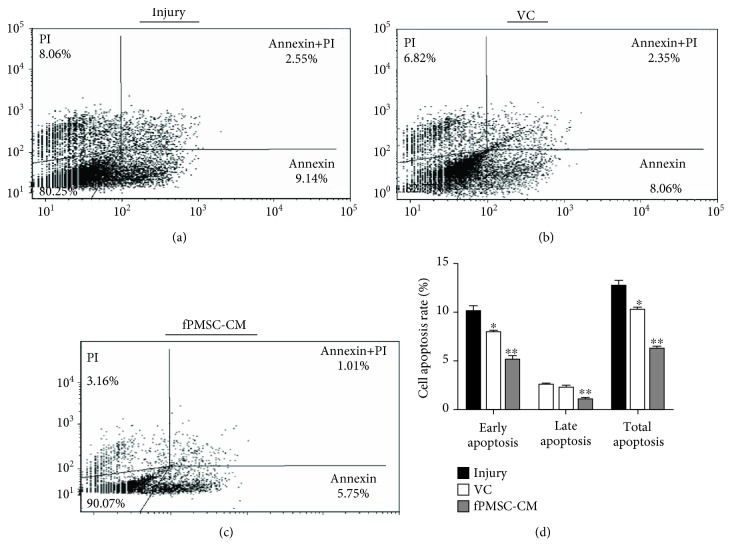
The inhibition of cell apoptosis of hfPMSC-CM in H_2_O_2_-induced cell injury. The A549 cells were injured with 600 *μ*mol/L H_2_O_2_ prior to being cultured in hfPMSC-CM, 100 *μ*mol/L VC, or media. Cell apoptosis was analyzed by a cytometric assay using an Annexin V-FITC/propidium iodide (PI) apoptosis detection kit. Fractions of apoptotic cells in A549 cells treated with normal media (a), 100 *μ*mol/L VC (b), and hfPMSC-CM (c) were presented. (d) The rate of apoptotic cells in (a–c). Data represented the mean ± SD from three independent triplicated experiments (*N* = 9, *t*-test). Compared with the injury group, ∗ and ∗∗ represent *p* < 0.05 and *p* < 0.01, respectively.

**Figure 4:**
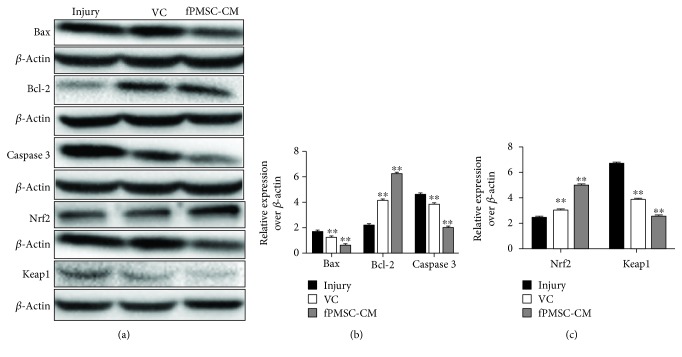
Immunoblotting analysis of the expression of apoptosis-related proteins. The A549 cells were injured with 600 *μ*mol/L H_2_O_2_ prior to being cultured in hfPMSC-CM, 100 *μ*mol/L VC, or control medium. (a) Immunoblotting assay determined the expression of Bax, Bcl-2, caspase 3, Nrf2, and Keap1 in injured A549 cells treated with VC or hfPMSCs-CM. (b) Semiquantitative analysis of the abundance of Bax, Bcl-2, and caspase 3 protein by densitometry assay. (c) Semiquantitative analysis of the expression of Nrf2 and Keap1 protein by densitometry assay. Data represented mean ± SD from three independent triplicated experiments (*N* = 9, *t*-test). Compared to the injury group, ∗ and ∗∗ represent *p* < 0.05 and *p* < 0.01, respectively.

In order to further investigate the potential impact of hfPMSC-CM on H_2_O_2_-induced cell oxidative injury, the A549 cells were treated with 600 *μ*mol/L H_2_O_2_ and cultured for 24 h. Then, the cells were exposed to hfPMSC-CM or medium containing 100 *μ*mol/L VC for 24 h. The flow cytometric analysis showed that VC and fPMSC-CM had no significant effect on the early apoptosis of A549 cells. As expected, both the VC and hfPMSC-CM groups could significantly protect cell apoptosis, especially in late apoptosis and total apoptosis, compared to the injury group (*p* < 0.01) (Figure 3). To further evaluate the underlying mechanism of apoptosis and antioxidative activity of hfPMSC-CM in injured A549 cells, the apoptosis-related proteins Bax, Bcl-2, caspase 3, and Nrf2-Keap1-ARE signaling pathway-related proteins Nrf2 and Keap1 were detected by immunoblotting analysis. Compared to the injury group, the expression of antiapoptotic protein Bcl-2 significantly increased, but the abundance of proapoptotic protein Bax (*p* < 0.01) and caspase 3 (*p* < 0.01) was significantly decreased in cells treated with VC and hfPMSC-CM, implying that VC and hfPMSC-CM could recover the injured A549 cells through a mechanism by inhibiting cell apoptosis. Importantly, the antioxidative protection and capacity of repair of hfPMSC-CM collected from P3 cell cultures to injured A549 cells were even superior to 100 *μ*mol/L of VC (*p* < 0.05) (Figures 4(a) and 4(b)). The Nrf2/Keap1/ARE signaling pathway-related proteins Nrf2 increased, but Keap1 decreased in the VC group and hfPMSC-CM group compared to the untreated injury group (Figures 4(a) and 4(c)) (*p* < 0.05), suggesting that VC and hfPMSC-CM were able to exert an antioxidative activity in part through the Nrf2/Keap1/ARE signaling pathway.

### 3.4. The hfPMSCs Protected the H_2_O_2_-Induced Cell Oxidative Injury by Regulating the Nrf2-Keap1-ARE Signaling-Mediated Cell Apoptosis in a Cell Coculture Model

**Figure 5:**
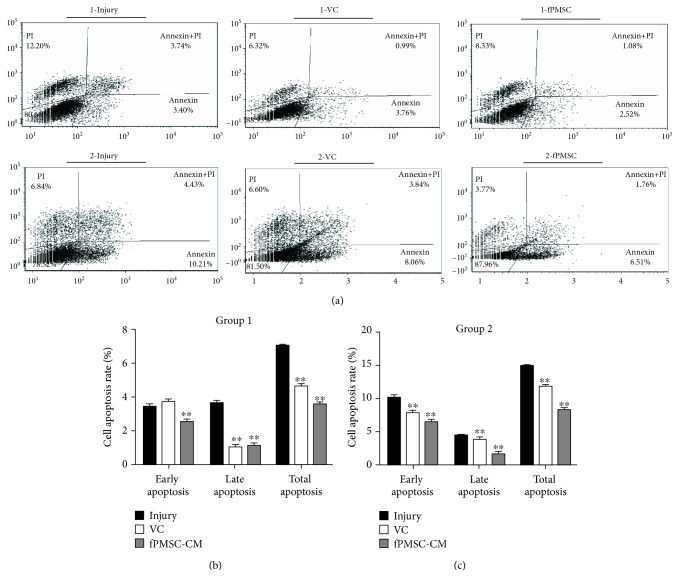
The protective role of hfPMSCs in A549 cells from cell apoptosis of H_2_O_2_-induced cell oxidative injury. The A549 cells were injured with 600 *μ*mol/L H_2_O_2_ prior to being treated with 100 *μ*mol/L VC or cocultured with hfPMSCs (3.0 × 10^6^/well) (group 1), or A549 cells (3.0 × 10^6^/well) were added with 100 *μ*mol/L VC or cocultured with hfPMSCs (3.0 × 10^6^/well) for 24 h, then the medium of the bottom was changed with 600 *μ*mol/L H_2_O_2_ for 24 h (group 2). The cell apoptosis was analyzed by a cytometric assay using an Annexin V-FITC/propidium iodide (PI) apoptosis detection kit. Fractions of apoptotic cells in A549 cells treated with normal medium, 100 *μ*mol/L VC, and hfPMSCs in group 1 (top panel) and in group 2 (bottom panel). (c, d) Analysis of apoptotic cell rates in (a), group 1 (c), and group 2 (d), respectively. Data represented the mean ± SD from three independent triplicated experiments (*N* = 9, *t*-test). Compared with the injury group, ∗ and ∗∗ represent *p* < 0.05 and *p* < 0.01, respectively.

**Figure 6:**
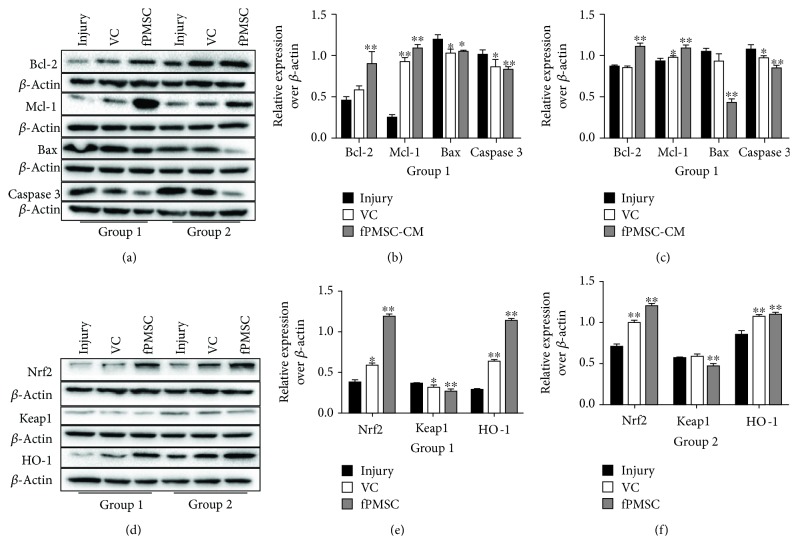
Immunoblotting analysis of cell apoptosis and Nrf2-Keap1-ARE signaling proteins. The A549 cells were injured with 600 *μ*mol/L H_2_O_2_ prior to being treated with 100 *μ*mol/L VC or cocultured with hfPMSCs (3.0 × 10^6^/well) (group 1), or A549 cells (3.0 × 10^6^/well) were added with 100 *μ*mol/L VC or cocultured with hfPMSCs (3.0 × 10^6^/well) for 24 h, then the medium of the bottom was exchanged with 600 *μ*mol/L H_2_O_2_ for 24 h (group 2). (a) Immunoblotting assay determined Bax, Bcl-2, caspase 3, and Mcl-1 expression. (b, c) Semiquantitative analysis for proteins of interest by densitometry assay in (a) both group 1 (b) and group 2 (c), respectively. (d) Immunoblotting assay determined Nrf2, Keap1, and HO-1 expression. (e, f) Semiquantitative analysis for proteins of interest by densitometry assay in (d) both group 1 (e) and group 2 (f), respectively. Data represented the mean ± SD from three independent triplicated experiments (*N* = 9, *t*-test). Compared to the injury group, ∗ and ∗∗ represent *p* < 0.05 and *p* < 0.01, respectively.

**Figure 7:**
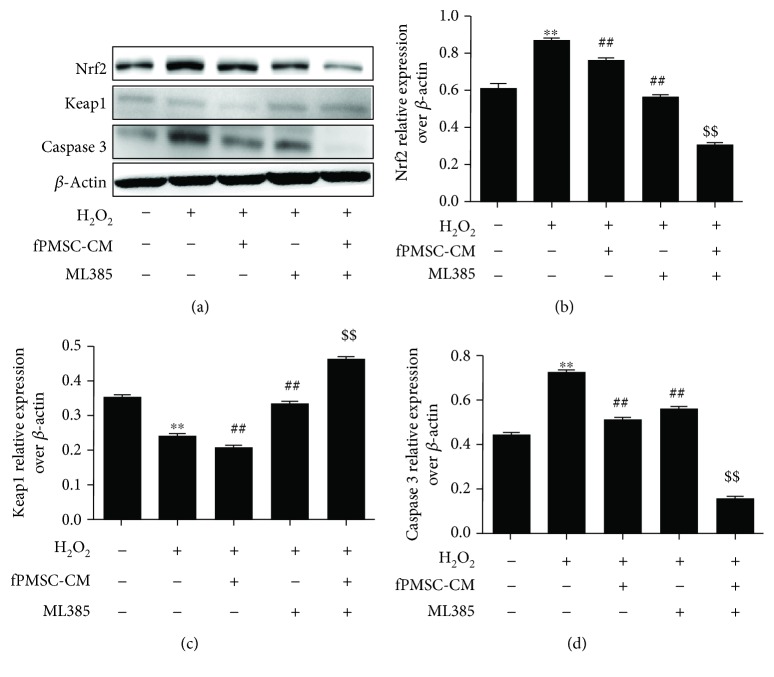
The inhibition of the Nrf2-Keap1-ARE signaling pathway led to an induction of cell apoptosis in H_2_O_2_-injured A549 cells. A549 cells (3.0 × 10^6^/well) were exposed to hfPMSC-CM (passage 3) or hfPMSC-CM collected from P3 cell cultures exposed to Nrf2 inhibitor ML385 for 24 h, prior to being treated with 600 *μ*mol/L H_2_O_2_ for 24 h. (a) Immunoblotting assay determined the abundance of caspase 3, Nrf2, and Keap1 proteins. (b–d) Semiquantitative analysis of proteins of interest by densitometry assay in (a). Data represented the mean ± SD from three independent triplicated experiments (*N* = 3, *t*-test). ^∗∗^ *p* < 0 01 vs. the control group; ^##^ *p* < 0 01 vs. the H_2_O_2_ group; ^$$^ *p* < 0 01 vs. the fPMSC-CM group.

In order to further explore the impact of hfPMSCs on the oxidative injury repair in A549 cells, a cocultured model with hfPMSCs seeded on the apical surfaces of Millicell inserts was used for investigation. As expected, the result of flow cytometric analysis showed that both VC and hfPMSCs could significantly reduce the fraction of cell apoptosis of A549 cells in the injury repair group (group 1) (Figure 5(a) top panel, and Figure 5(b)) or protective injury group (group 2) (Figure 5(a) bottom panel, and Figure 5(c)). Immunoblotting assay further identified that both VC and hfPMSCs strikingly inhibited cell apoptosis by inhibiting the expression of Bax and caspase 3 but inducing the expression of Bcl-2 and Mcl-1 in both injury repair group (group 1) (Figures 6(a) and 6(b)) and injury protection group (group 2) (Figures 6(a) and 6(c)), respectively. In addition, the roles of hfPMSCs in the Nrf2/Keap1/ARE signaling pathway underlying oxidative injury protection were further interrogated. An addition of VC or a coculture of hfPMSCs significantly induced the accumulation of Nrf2 and subsequently decreased the expression of Keap1, a negative regulator of Nrf2 activity, compared to the untreated injury group (Figures 6(d)–6(f)). It has been well documented that oxidative stress could induce an activation of Nrf2, which in turn promotes the transcriptional activation of HMOX1, a gene coding for the cytoprotective and stress-responsive HO-1 protein. Here, the expression of HO-1 was also clearly induced in both VC group and hfPMSC group compared to the untreated injury group (Figures 6(d)–6(f)). In order to further investigate whether the Nrf2 inhibitor ML385 could inhibit the antioxidative capacity of hfPMSC-CM, the inhibitory effect of Nrf2 inhibitor ML385 on hfPMSC-CM-mediated antioxidant activity was evaluated in CMs from P3-P6 cell cultures. Unexpectedly, the result revealed that hfPMSC-CM collected from passage 3 cell cultures exposed to Nrf2 inhibitor ML385 had no effect on antioxidative capacity of hfPMSC-CM on the scavenging of DPPH, O2^−^, ·OH, SOD, and GSH-PX (data not shown), despite that ML385 could significantly inhibit Nrf2/Keap1/ARE signaling activity (Figures 7(a)–7(c)) and caspase 3 expression (Figures 7(a) and 7(d)) in A549 cells. These data suggested that both hfPMSCs and hfPMSC-CM could protect the H_2_O_2_-induced cell oxidative injury at least in part by regulating the Nrf2-Keap1-ARE signaling-mediated cell apoptosis; however, the precise mechanism needs to be further interrogated.

## 4. Discussion

Mesenchymal stem cells (MSCs) have been used as potential clinical cell therapeutic applications, which exhibit potencies of multiple differentiation and immune regulation. They can secrete some immune factors such as IL-10, interferon *γ* (IFN-*γ*), and IL-4 and participate in modulating the immunological response, promoting an anti-inflammatory or tolerant phenotype [22–24]. MSCs also show ability to repair injuries through mechanisms by differentiating into multiple cell types/lineages [22] and releasing a wide range of factors such as cytokines, chemokines, and growth factors [25]. Therefore, cell-free therapy with MSC secretome has been considered as a novel potential approach of cell therapy for many diseases [26, 27].

In this study, we attempted to seek the antioxidative capacity of hfPMSC-CM during *in vitro* expansion culture in a serum-free culture condition and further analyze the potential biological functions of hfPMSCs-CM and hfPMSCs on the H_2_O_2_-induced A549 cell oxidative injury. Our results indicated that the hfPMSC-CM has the total antioxidative capacity (T-AOC), with scavenging ability of free radicals DPPH, hydroxyl radical (·OH), and superoxide anion radical (O_2_-), as well as activities of antioxidant enzymes of SOD and GSH-PX. Of note, a significantly higher antioxidative capacity was found in the hfPMSC-CM of passage 3 (P3) and P4 cells. This might be reasoned that when cells were expanded at P3 and P4, fibroblasts or epidermal cells were senescent and only MSCs could survive at this point as our previous findings [17–19]. In addition, MSCs started to differentiate into other cell types and the proliferation slowly down in subsequent passages in this culture condition (data not show), partially due to genetic and/or epigenetic alterations of hfPMSC (from passage 3 to passage 8) during long-term culture under the serum-free condition [15]. Intriguingly, the hydroxyl radical (·OH) inhibiting ability had no significant difference in all examined passages. Therefore, in this study the P3 hfPMSCs were chosen for all experiments.

The main aim of this study was to investigate whether the hfPMSC-CM has the protective capacity to prevent H_2_O_2_-induced oxidative injury of A549 alveolar epithelial cells. Many studies have demonstrated that MSCs repair damaged organs or injury cells by secreting cytokines and growth factors [28, 29]. In this respect, MSCs could alleviate acute lung injury in rats caused by lipopolysaccharide (LPS) and reduce oxidative stress injury in rats [14]. MSCs also showed potentials to protect cells from oxidative stress-associated injury [30]. In this context, we found that a conditioned medium of hfPMSCs could inhibit cell apoptosis in A549 cells. When oxidative stress-damaged A549 cells were cultured in a conditioned medium of hfPMSCs or cocultured with hfPMSCs , the abundance of proapoptotic proteins Bax and caspase 3 was decreased, while the expression of antiapoptotic proteins Bcl-2 and Mcl-1 was increased. This result clearly indicated that hfPMSCs were able to protect A549 cells from oxidative injury by inhibiting oxidative stress-induced cell apoptosis.

Nuclear factor erythroid 2-related factor 2 (Nrf2) is a key transcription factor, which has been identified to be able to activate antioxidative reactions in 1994 by Moi et al. [31]. Furthermore, recent studies also found that Nrf2 signaling was a key pathway that was essential for the antioxidant enzyme to remove ROS and other harmful substances and protect the body from oxidative stress [32–34]. In this context, the upregulation of many antioxidative enzymes or inhibition of lipid peroxidation was mediated by Nrf2 [35, 36]. When the oxidative injured A549 cells were treated with a conditioned medium of hfPMSCs, the expression of Nrf2 was significantly increased, but the Kelch domain of kelch-like ECH-associated protein 1 (Keap1), a signaling molecule binding to motifs in the N-terminal region of Nrf2, was decreased. In addition, Nrf-2 also regulated the expression HO-1 [37]. Our results also showed that hfPMSC-CM or hfPMSCs could promote HO-1 expression in oxidative injury of A549. These results demonstrated that the conditioned medium of hfPMSCs could activate the Nrf-2 signaling pathway and protect the cells from oxidative injury. Consequently, many signaling pathways, such as PI3/AKT/ERK pathways, have been reported to regulate HO-1 [37, 38]. Therefore, further studies still need to investigate signaling pathways playing key roles in preventing H_2_O_2_-induced oxidative injury in primary lung epithelial cells *in vitro* and animal models *in vivo*.

## 5. Conclusion

In the present study, we interrogated the antioxidative capacity of hfPMSCs to protect alveolar epithelial cells from H_2_O_2_-induced injury. Our results demonstrated a considerable antioxidative ability of hfPMSC-CM and hfPMSCs to scavenge free radicals and protect A549 cells from H_2_O_2_-induced injury. Mechanistically, both hfPMSC-CM and hfPMSCs were capable of inhibiting oxidant-induced cell apoptosis in part by regulating Nrf2/Keap1/ARE signaling-mediated cell apoptosis. This study thus suggests that the antioxidative potency of hfPMSCs may be translated into clinic for immunoregulation and antioxidant treatments of oxidative stress diseases, which warrants for further investigation.

## Data Availability

All data generated or analyzed during this study are included in this published article.

## Abbreviations

ALI: Acute lung injury carcinoma
ARDS: Acute respiratory distress syndrome
ARE: Antioxidant response element
Bax: BCL2-associated X
Bcl2: B cell lymphoma-2
BM: Bone marrow
BMSCs: Bone marrow mesenchymal stem cells
CM: Conditioned medium
ECL: Enhanced chemiluminescence
EVs: Extracellular vesicles
hfPMSCs: Human fetal placental mesenchymal stem cells
HGF: Hepatocyte growth factor
HO-1: Glutathione peroxidase
keap1: Kelch-like ECH-associated protein 1
Nrf2: Nuclear factor erythroid 2-related factor 2
MSCs: Mesenchymal stem cells
PVECs: Pulmonary microvascular endothelial cells
ROS: Reactive oxygen species
SLE: Systemic lupus erythematosus
T-AOC: Total antioxidative capacity.

## Supplementary Materials

Supplementary Materials Supplementary Figure 1: standard curve describing the total antioxidant capacity of vitamin C (*n* = 3) (A) and the percentage of different passage hfPMSC-conditioned media of T-AOC vs. control group (B). Data represented the mean ± SD from three independent triplicated experiments (*N* = 9, ANOVA). ∗ and ∗∗ represent *p* < 0.05 and *p* < 0.01, respectively.
