# Supplementary figures and images for: Nrf2/Keap1/ARE Signaling Mediated an Antioxidative Protection of Human Placental Mesenchymal Stem Cells of Fetal Origin in Alveolar Epithelial Cells
Source: Oxid Med Cell Longev. 2019 May 14;2019:2654910. doi: 10.1155/2019/2654910 (PMC6537011; doi:10.1155/2019/2654910)

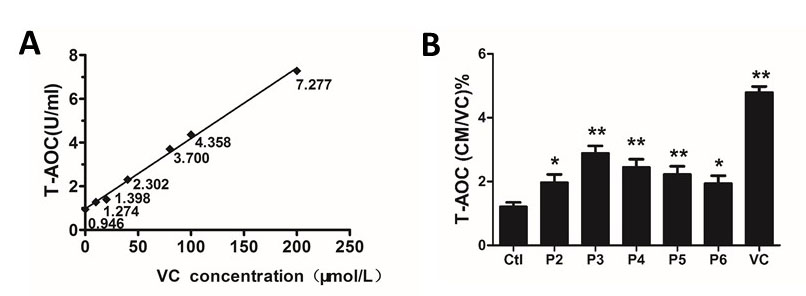

Supplement: Supplementary Materials — Supplementary Figure 1: standard curve describing the total antioxidant capacity of vitamin C (n = 3) (A) and the percentage of different passage hfPMSC-conditioned media of T-AOC vs. control group (B). Data represented the mean ± SD from three independent triplicated experiments (N = 9, ANOVA). ∗ and ∗∗ represent p < 0.05 and p < 0.01, respectively. [file 2654910.f1.jpg]
